# Anterior segment OCT for imaging PAUL^®^ glaucoma implant patch grafts: a useful method for follow-up and risk management

**DOI:** 10.1007/s00417-024-06708-2

**Published:** 2024-12-16

**Authors:** Pascal Schipper, Constance Weber, Ke Lu, Siqi Fan, Verena Prokosch, Frank G. Holz, Karl Mercieca

**Affiliations:** 1 Department of Ophthalmology, University Hospital Bonn, Ernst-Abbe-Str. 2, 53127 Bonn, Germany; 2 Department of Ophthalmology, University Hospital Cologne, Cologne, Germany

**Keywords:** PAUL^®^ glaucoma implant, Glaucoma drainage devices, Glaucoma surgery, Patch grafts, Anterior segment OCT, Tube erosion

## Abstract

**Aim:**

To evaluate a useful, non-contact method for the follow-up of pericardium patch graft changes in patients undergoing PAUL^®^ Glaucoma Implant (PGI) surgery using high-resolution anterior segment optical coherence tomography (OCT) to predict tube erosions.

**Methods:**

Prospective analysis over six months of tube pericardium patch graft thickness of PGI surgical cases at the University Eye Hospital Bonn, Germany, from November 2021 to August 2022. In all eyes, Tutopatch^®^ (RTI Surgical, United States) pericardium was used to cover the implant intra-operatively. Anterior segment OCT (AS-OCT, Heidelberg ANTERION^®^ Swept-Source-OCT) examinations were performed following a standardized protocol to measure quantitative and qualitative aspects of the patch grafts before surgery, and at three and six months after surgery.

**Results:**

Twenty-six eyes of 26 patients were included. Thickness of the patch material was 1188 µm (IQR 415 µm) directly after implantation and decreased over time to 1068 µm (IQR 478 µm) at 3 months and 846 µm (IQR 677 µm) at 6 months. No significant differences between groups were shown concerning gender (*p* = 0.128), ethnicity (*p* = 1.000), age (*p* = 0.741), glaucoma type (*p* = 0.173), other concurrent diseases (*p* = 0.302), former glaucoma surgeries (*p* = 1.000) and the quadrant of implantation (*p* = 0.555). Five eyes developed implant exposure. When comparing eyes with and without tube exposure, no significant differences were shown in average patch thickness above the tube directly after implantation (*p* = 0.476). However, significant differences in average thickness were observed at 3 months (*p* = 0.013) and 6 months (*p* = 0.005).

**Conclusions:**

Pericardial patch grafts tend to thin over time which can be assessed by AS-OCT, the latter proving to be a useful method to follow-up patients who undergo patch graft implantation during PGI surgery. This investigation could potentially help identify patients at risk of tube exposure which in turn could lead to modification of patient management. It could also possibly be used in future studies to find more suitable patch materials.

## Abstract (highlights)

****Key messages**:**

*****What is known?***:**

- Anterior segment optical coherence tomography (AS-OCT) can be used to image filtering blebs after glaucoma surgery.

*****What is new?***:**

- AS-OCT can be used to quantify patch grafts used in PAUL^®^ glaucoma implant surgery.
- Patients at risk for tube exposure after glaucoma implant surgery can be successfully monitored using AS-OCT.

## Introduction

Glaucoma surgical options have evolved rapidly over the past decade and glaucoma drainage implant (GDI) or ‘tube’ surgery is becoming an increasingly popular choice, particularly in eyes with secondary glaucoma (e.g. uveitic, neovascular) or with significant conjunctival scarring, e.g. following pars plana vitrectomy or failed previous bleb-forming surgery (e.g. trabeculectomy) or as a primary intervention in open angle glaucoma [1]. The Ahmed^®^ glaucoma valve (AGV, New World Medical, United States), the Baerveldt^®^ tube (BVT, Abbott Medical Optics, United States), the Molteno^®^ tube (Molteno Ophthalmic Limited, New Zealand) and the PAUL^®^ glaucoma implant (PGI, Advanced Ophthalmic Innovations, Singapore) are commonly used GDIs [2]. Besides risks in common with trabeculectomy, such as hypotony, bleb scarring, suprachoroidal haemorrhage and endophthalmitis [3, 4], GDIs also have additional risks such as diplopia, corneal decompensation and implant exposure, with the latter often leading to the need for revision surgery [5]. A common method of reducing implant exposure rates is the use of allogeneic tissue patch grafts which are secured over the tube during surgery.

There is currently a lack of studies which describe accurate visualization of these patch grafts, and we could only identify one study in the literature investigating patch grafts in tube surgery. The latter was conducted exclusively with the AGV and showed a reduction of patch material thickness over time [6]. However, to the best of our knowledge, there are no studies with regards to the use of AS-OCT for morphological analysis of patch materials in the context of PGI surgery, particularly for the purpose of assessing and/or predicting tube erosion. This publication demonstrates the possible advantages of using AS-OCT for follow-up of patients after PGI surgery.

## Material and methods

### Patients

Patient recruitment for this study took place from November 2021 to August 2022 at the University Eye Hospital Bonn, Germany.

Examinations were carried out pre-operatively, within the first post-operative week, three months and six months after surgery using high-resolution AS-OCT (Heidelberg ANTERION^®^ Swept-Source OCT, Heidelberg Engineering GmbH, Germany) to visualize and measure the inserted patch material. In addition to this prospective analysis of the patch materials, further patient data was collected retrospectively from the patient medical records. These included best corrected visual acuity (BCVA) using Snellen table and then converted to logMAR for statistical analysis; IOP, measured using Goldmann applanation tonometry; slit lamp and fundus examinations. Furthermore, demographic information such as patient age, gender and ethnic origin, glaucoma type and any previous glaucoma surgery, the number of eye drops and other systemic diseases (e.g. diabetes mellitus, systemic hypertension) and the use of oral anticoagulants were recorded.

**Fig. 1:**
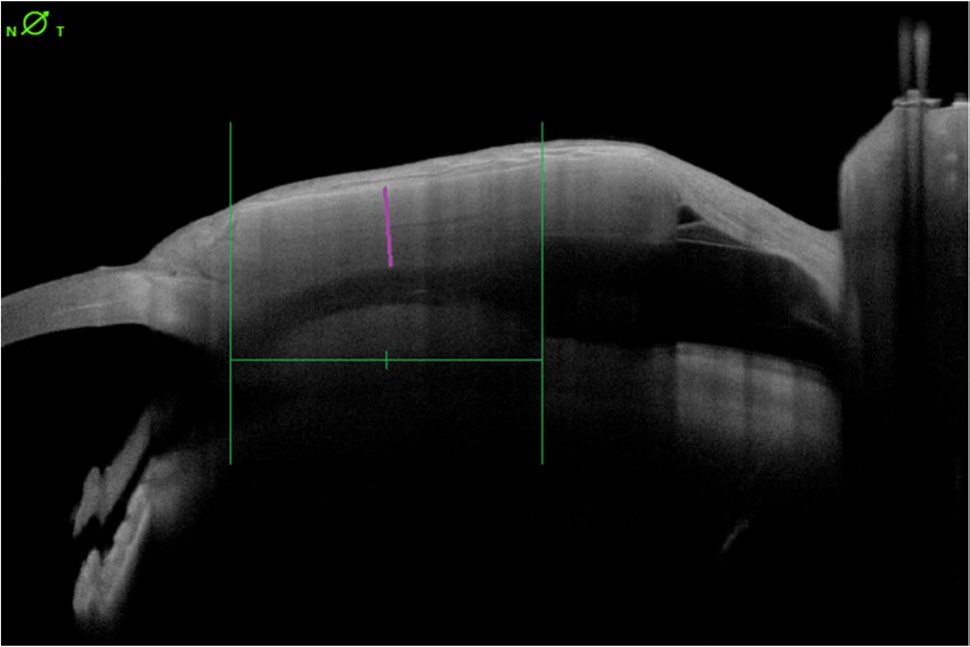
Example of measurement of patch material thickness above the tube. The middle of the distance between the insertion point in the anterior chamber (left long vertical line) and the beginning of the plate of the glaucoma implant (right long vertical line) is used for reference. Above this point (short vertical line) the patch material is measured (oblique line). The measurement unit is pixels and is then converted to µm

The AS-OCT images were taken by trained personnel according to precisely defined parameters. For each series of images, 25 OCT scans with a length of 16.0 mm and a height of 7.5 mm were performed at a 45° or 135° angle, depending on which eye (right or left) and which quadrant was used for implantation. All examinations were performed on one AS-OCT device. The images were then measured using the Heidelberg Eye Explorer platform's own evaluation application (HEYEX2). For this purpose, the centre between the entry point of the tube into the anterior chamber and the beginning of the PGI plate was determined and the patch material thickness measured in pixels above it (see Fig. 1). The thickness measurements were then taken using three OCT slices above and three OCT slices below the tube to calculate an average patch thickness from the values. The procedural instructions and measurement techniques have undergone internal validation to ensure that the OCT images and measurements are examiner-independent and repeatable. Both the intra-rater reliability and the inter-rater reliability for the measurement protocol used were accessed via a sub-sample to check for reliability. The intraclass correlation coefficient (ICC) was very high for both the intra-rater reliability at 0.990 [0.962–0.997] and for the inter-rater reliability at 0.976 [0.901–0.993].

The determined values for patch graft thickness were multiplied by a device and tissue specific conversion factor of 6.82 to obtain patch material thickness in µm. The conversion factor was determined using standardized thickness measurements via Pentacam^®^ (OCULUS Optikgeräte GmbH, Germany) and standardized measurements by AS-OCT in relation to the non-standardized measurements in pixels by dividing the standardized value of 526.5 µm averaged from several measurements by the value of 77.25 in pixels averaged from several AS-OCT measurements at the same location.

The study was approved by the ethics committee of the University Hospital Bonn and all patients signed a specific informed consent form before participating in the study. The study therefore complies with the criteria of the Declaration of Helsinki.

### Surgical technique

During PGI implantation, sponges soaked with mitomycin C (MMC) at a concentration 0,5 mg/ml were applied in all patients for two minutes after formation of a fornix-based conjunctival/Tenon’s pocket via a limbal incision and identification of the relevant extraocular musculature in the area. After suture fixation of the plate with 9.0 nylon, and insertion of the tube, partially occluded with a 6.0 intraluminal prolene suture, into the anterior chamber using a two-step scleral tunnel technique, the tube was secured with a 9.0 mattress suture and then covered with patch material. The patch material had a width of approximately 6 mm and a length of 6 to 8 mm, which was sufficient to cover the entire length of the tube from the plate edge to 1 mm anterior to the insertion point in the sclera. A double-layered patch material, Tutopatch^®^ pericardium (RGI Surgical, United States), was used in all patients and was secured with TISSEEL (Baxter, United States) two-component adhesive. The conjunctiva was subsequently closed with 10.0 nylon. The full surgical technique used routinely in our unit is detailed in the publication by Vallabh et al. from 2022 [7].

### Statistical analysis

Statistical analyses were performed using SPSS Statistics Version 27.0.0 (IBM Corporation, New York). For the nominal variables gender, ethnicity, glaucoma type, other eye diseases, diabetes, arterial hypertension, use of anticoagulants, lens status, status after vitrectomy, number of eye drops preoperatively, status after former glaucoma surgery, the tube localisation and the implantation quadrant, as well as the assessments of the presence of a fluid layer between the individual sheets of patch material and the patch material excess over the limbus Fischer's exact test was performed. After checking for normal distribution using the Shapiro–Wilk test, for the numerical variables age, pre-operative visual acuity (logMAR) and pre-operative IOP, as well as the various thickness measurements of the conjunctiva and patch materials, the Mann–Whitney U test was used due to the results of the Shapiro–Wilk test. Results were considered statistically significant at a P-value of less than 0.05.

## Results

Twenty-six patients who underwent PGI surgery with Tutopatch^®^ pericardium were included in the study. 15 patients were male, and 11 patients were female. The mean age was 64.8 years (26–81 years). 12 patients had open-angle glaucoma, 10 patients had secondary glaucoma, and 4 patients had a different type of glaucoma. 11 patients had a history of pars plana vitrectomy, and 13 patients had had another glaucoma operation in the past. 5 patients developed tube exposure with subsequent need for revision surgery. Further and more detailed patient data are shown in Table 1.

**Table 1:** Patient demographics and pre-operative data

Variable	Results (*n* = 26)
Gender (male | female)	15 | 11
Age (mean | median | range) (years)	64.8 | 68.5 | 26—81
Ethnicity (Caucasian | other)	25 | 1
Glaucoma type:	
Primary open-angle glaucoma	12
Angle-closure glaucoma	1
Pseudoexfoliation glaucoma	2
Pigmentary glaucoma	1
Secondary glaucoma	10
Mean visual acuity (logMAR)	0.72 ± 0.80
Mean intraocular pressure (mmHg)	24.0 ± 7.8
Diabetes (yes | no)	5 | 21
Arterial hypertension (yes | no)	11 | 15
Anticoagulation (yes | no)	3 | 23
Lens status (phakic | pseudophakic)	4 | 22
Post-vitrectomy (yes | no)	11 | 15
Number of eye drops (0 | 1 | 2 | 3 | 4)	0 | 0 | 2 | 10 | 14
Oral acetazolamide (yes | no)	8 | 18
Former glaucoma surgeries (yes | no):	13 | 13
Trabeculectomy	4
Cyclophotocoagulation	3
Canaloplasty	3
Trabectome	3
Tube localisation (anterior chamber | pars plana)	25 | 1
Implantation quadrant (superotemporal | superonasal)	22 | 4
Tube exposure (yes | no)	5 | 21

**Fig. 2:**
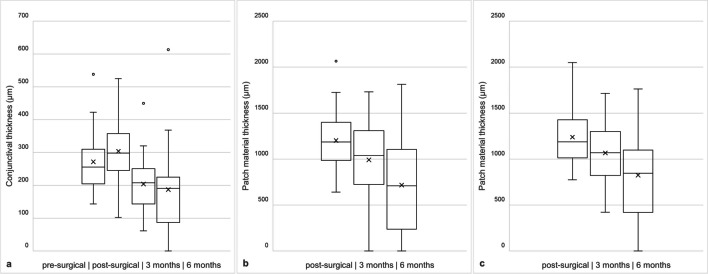
Boxplots displaying the development of the thickness of the conjunctiva (**a**) and the patch materials over the tube (**b**) and the overall patch material thickness (**c**) over time. Circle = outlier, cross = mean

**Fig. 3:**
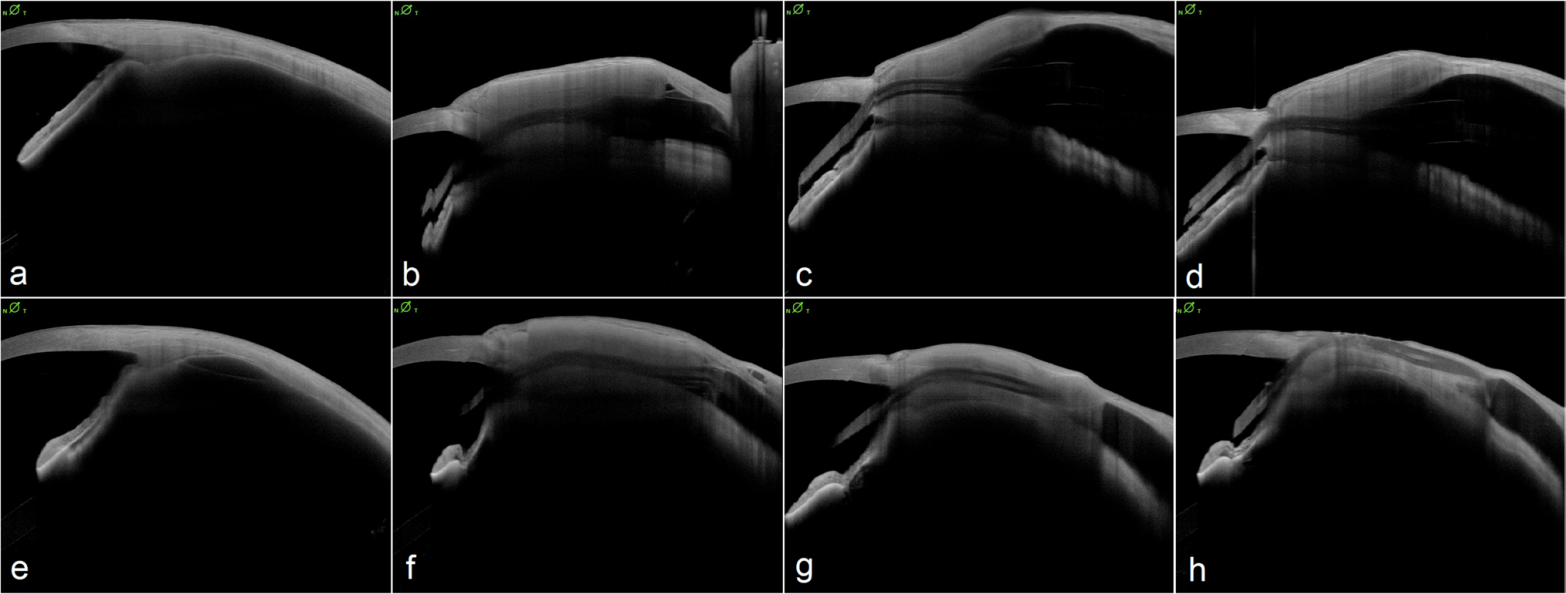
Patient 1 (a—d) with repetitive high-resolution anterior segment OCT imaging pre-operative (**a**), directly post-operative (**b**), after 3 months (**c**) and after 6 months (**d**). Throughout the examinations no tube exposure could be detected. Patient 2 (e—h) with repetitive high-resolution anterior segment OCT imaging pre-operative (**e**), directly post-operative (**f**), after 3 months (**g**) and after 6 months (**h**). After 6 months a tube exposure was detected, and the patient planned for revision surgery. Scan pattern details: 25 B-scans, 8 scans used for averaging, 45° B-scan angle, 7.50 mm B-scan height, 16.00 mm B-scan length

As shown in Fig. 2, the conjunctival thickness changed from 256 µm (IQR 106 µm) pre-operatively to 298 µm (IQR 112 µm) immediately post-operatively, 208 µm (IQR 107 µm) at three months and 191 µm (IQR 138 µm) at six months after surgery. The thickness of the patch material directly over the tube decreased from initially 1186 µm (IQR 416 µm) to 1039 µm (IQR 584 µm) at three months and 709 µm (IQR 869 µm) at six months after surgery. Similarly, the total thickness of the patch material changed from initially 1188 µm (IQR 415 µm) to 1068 µm (IQR 478 µm) at three months and 846 µm (IQR 677 µm) at six months after surgery. Figure 3 shows the examinations of two patients over time, one without tube erosion and one with tube erosion. While 20 patients still showed a thin layer of fluid between the individual sheets of patch material at the beginning, this was still the case in 8 patients after three months and in only 1 patient after six months. A similar trend was seen in the excess of patch material, that extends beyond the limbus. Initially, 11 patients showed an excess, after three months still 7 patients and after six months only 2 patients.

**Fig. 4:**
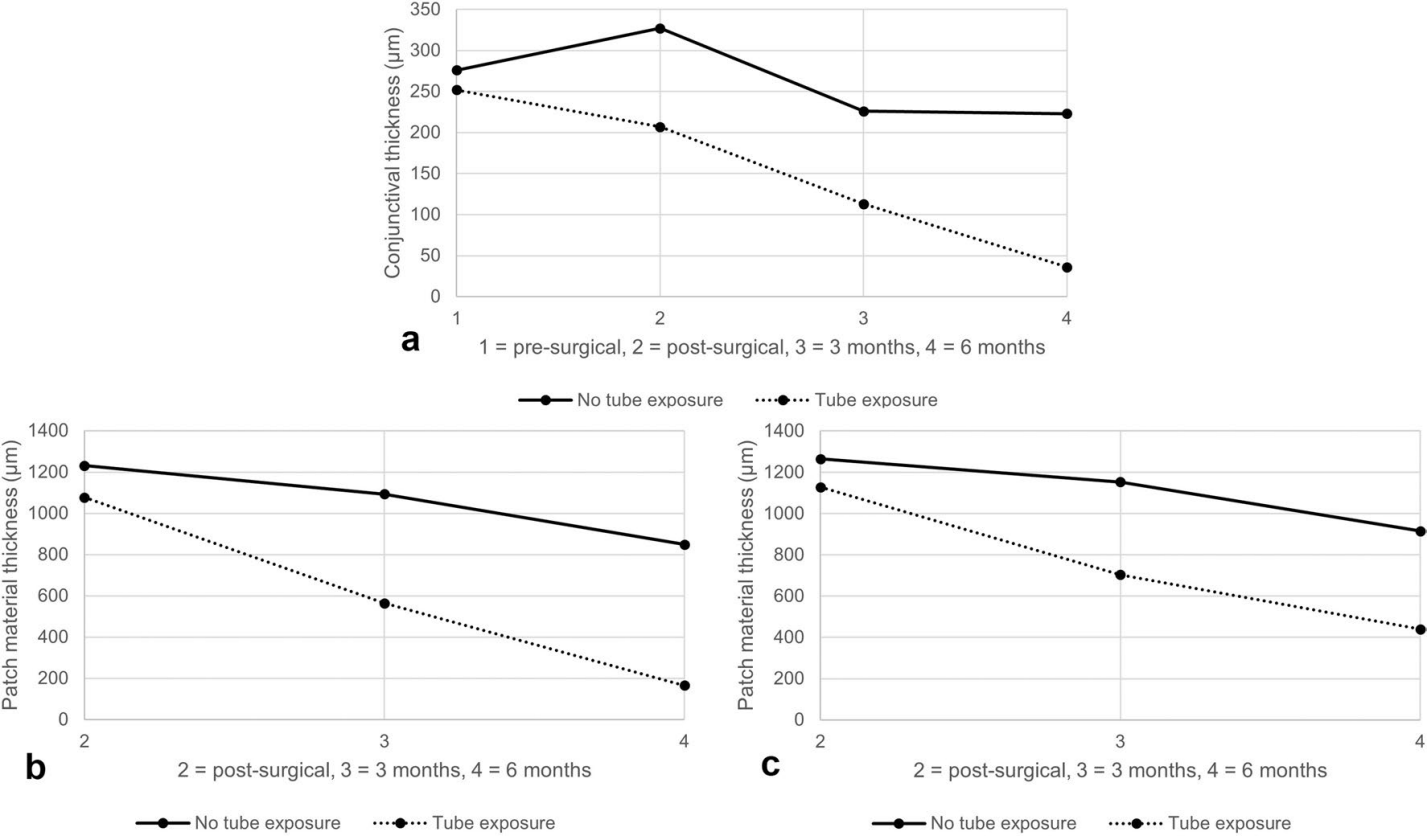
Development of the thickness of the conjunctiva and the patch materials over time. The thickness of the conjunctiva (**a**), the thickness of the patch material over the tube (**b**) and the overall thickness of the patch material (**c**) decreases over time. In the group comparison, all three graphs show a clear difference already after three months with lower thicknesses in the group with tube exposure

Table 2 details the comparison of patients with versus without tube exposure for gender, ethnicity, glaucoma type, other eye diseases, diabetes, arterial hypertension, use of anticoagulants, lens status, post-vitrectomy status, number of pre-operative eye drops, status of previous glaucoma surgery, tube localisation and implantation quadrant, as well as assessing the presence of a fluid layer between the individual sheets of patch material and the patch material remnants over the limbus. Significant differences (*p* < 0.05) were shown for lens status (*p* = 0.014). The status post-vitrectomy was just barely non-significant (*p* = 0.053). All other nominal variables showed no significant difference. There were no significant differences in age (*p* = 0.741), preoperative visual acuity (*p* = 0.603), preoperative IOP (*p* = 0.622) and preoperative conjunctival thickness (*p* = 0.987). Post-operatively, the conjunctival thickness showed a significant difference (*p* = 0.024). The measured patch material parameters showed no significant difference. After three months, there were significant differences in conjunctival thickness (*p* = 0.002), patch material thickness over the tube (*p* = 0.013) and total thickness of the patch material (*p* = 0.012). After six months, the conjunctival thickness (*p* < 0.001) and the patch material thickness over the tube (*p* = 0.005) were still significantly different. The total thickness of the patch material (*p* = 0.067) was no longer significantly different. The exact data are shown in Table 2 and Fig. 4 shows the reduction of the conjunctival thickness and the patch material’s thicknesses over time.

**Table 2:** Group statistics and comparison between patients without tube exposure and patients with tube exposure

Variable	No tube exposure (*n* = 21)	Tube exposure (*n* = 5)	*P*-value
Gender (male | female)	14 | 7	1 | 4	0.128^a^
Ethnicity (Caucasian | other)	20 | 1	5 | 0	1.000^a^
Age (years)	64.2 ± 13.3	67.4 ± 12.9	0.741^b^
Glaucoma type (POAG | ACG | XFG | PG | SG)	11 | 1 | 1 | 0 | 8	1 | 0 | 1 | 1 | 2	0.173^a^
Concurrent eye diseases (e.g. uveitis, high myopia, retinal vein occlusion) (yes | no)	15 | 6	2 | 3	0.302^a^
Diabetes (yes | no)	4 | 17	1 | 4	1.000^a^
Arterial hypertension (yes | no)	8 | 13	3 | 2	0.620^a^
Anticoagulation (yes | no)	1 | 20	2 | 3	0.085^a^
Lens status (phakic | pseudophakic)	1 | 20	3 | 2	0.014^a^
Post-vitrectomy (yes | no)	11 | 10	0 | 5	0.053^a^
Former glaucoma surgery (yes | no)	10 | 11	3 | 2	1.000^a^
Number of eye drops (0 | 1 | 2 | 3 | 4)	0 | 0 | 2 | 6 | 13	0 | 0 | 0 | 4 | 1	0.147^a^
Pre-operative:			
Visual acuity (logMAR)	0.82 ± 0.86	0.32 ± 0.19	0.603^b^
Intraocular pressure (mmHg)	23.5 ± 7.9	26.0 ± 8.0	0.622^b^
Conjunctival thickness (µm)	276 ± 99	252 ± 28	0.987^b^
Post-operative:			
Tube localisation (anterior chamber | pars plana)	20 | 1	5 | 0	1.000^a^
Implantation quadrant (superotemporal | superonasal)	17 | 4	5 | 0	0.555^a^
Conjunctival thickness (µm)	327 ± 83	207 ± 95	0.024^b^
Mean patch material thickness above the tube (µm)	1232 ± 294	1077 ± 338	0.476^b^
Mean overall patch material thickness (µm)	1265 ± 301	1129 ± 340	0.380^b^
3 months after surgery:			
Conjunctival thickness (µm)	226 ± 74	113 ± 46	0.002^b^
Mean patch material thickness above the tube (µm)	1094 ± 382	564 ± 398	0.013^b^
Mean overall patch material thickness (µm)	1153 ± 318	704 ± 260	0.012^b^
6 months after surgery:			
Conjunctival thickness (µm)	223 ± 107	36 ± 34	< 0.001^b^
Mean patch material thickness above the tube (µm)	849 ± 518	166 ± 210	0.005^b^
Mean overall patch material thickness (µm)	916 ± 446	440 ± 405	0.067^b^

*POAG* primary open-angle glaucoma; *ACG* angle-closure glaucoma; *XFG* pseudoexfoliation glaucoma; *PG* pigmentary glaucoma; *SG* secondary glaucoma

For numerical variables Mann–Whitney U test was used and for ordinal and categorical variables Fisher's exact test was performed

^a^ Fisher's exact test

^b^ Mann–Whitney U test

The time intervals between the patch material measurements and the surgery showed no significant group difference at all time points.

## Discussion

In this prospective study, patients after PGI implantation were examined using high-resolution AS-OCT. A variety of imaging techniques have already been utilized to analyse filtering blebs after several glaucoma procedures. Ultrasound biomicroscopy (UBM) has been used to evaluate subconjunctival drainage after deep sclerectomy [8] and magnetic resonance imaging (MRI) of filtering blebs after BVT implantation has been used to visualize morphological and functional differences between cases [9]. AS-OCT has also been used for imaging blebs, e.g. after trabeculectomy, providing targeted, accurate measurements of bleb parameters and visualizing changes not usually discernible on slit lamp biomicroscopy [10, 11]. The advantages of AS-OCT (e.g. measurement accuracy and comparability) were also shown with regards to bleb morphology, e.g. by enabling visualization and quantification of bleb vascularity [12]. Chelerkar and colleagues were able to show that AS-OCT allows accurate morphological assessment of filtering blebs and used that data for a group comparison of patients undergoing phacotrabeculectomy with either MMC or Ologen™ (AEON Astron Europe B.V., Netherlands) as an anti-scarring agent [13]. Another study by Jung et al. showed the benefit of AS-OCT for analysing blebs after AGV implantation [14]. However, all of these studies examined the bleb morphology itself and did not perform patch grafts measurements apart from the afore-mentioned study by Akbas et al. [6] which did mention the benefit of AS-OCT for imaging patch grafts in the case of AGV implantation. This study also revealed that thinning of patch material was greatest over the tube itself.

In comparison to studies with ultrasound examinations of filtering blebs, which can lead to bleb compression and thus influence measurements, AS-OCT is a non-contact method. Due to the differences in tube thickness and plate configuration between the AGV and the PGI (e.g. PGI having a smaller inner and outer cross sectional diameter) [15] we decided to perform an analysis of patch grafts after PGI surgery and used AS-OCT rather than UBM or MRI because of the advantages mentioned above. Our study shows that patch material thickness decreases progressively over time, with an initial thickness of 1186 µm (IQR 416 µm) reducing to 709 µm (IQR 869 µm) after six months. While the patch thinning progressed slowly in most patients, a few patients developed rapid progression with resulting tube exposure and consequent need for revision surgery. When analysing preoperative patient characteristics, there seems to be no clear preoperative risk factor that would allow differentiation between patients at risk and those not at risk, as only lens status showed a statistically significant difference regarding preoperative factors. There is no plausible explanation for the latter finding however, and it may well be related to the small sample size. Furthermore, we have not found studies with larger cohorts demonstrating any significant correlation between lens status and tube erosion risk. A study by Trubnik et al. actually suggests the contrary, with pseudophakia being a risk factor for erosion, although no distinction was made between cataract surgery and other intraocular surgery in their statistical analysis [16]. If no obvious pre-operative risk factors for tube erosion are present (e.g. clinically visible thin conjunctiva) the ability to identify tubes at risk of erosion becomes more significant and it would be useful to identify postoperative factors or parameters which could differentiate between low-risk and high-risk patients.

The analysis of the various thickness measurements shows the importance of conjunctival thickness as a risk factor for tube erosion with a significant group difference concerning this measure (*p* = 0.024) already being evident during the first few days after surgery. Thin conjunctiva in the first post-operative days should therefore raise the alert level, whereas in this period the actual patch thickness does not seem to distinguish which eyes are at higher risk of erosion. Similar patch thicknesses in both groups also ensured group comparability, with no bias based on this factor. Statistically significant group differences with regard to patch material thickness were shown at three months both in terms of thickness directly over the tube (*p* = 0.013) and total thickness (*p* = 0.012). This finding shows that it is possible to monitor patients for rapid patch thinning at the 3-month mark and advise closer follow-up either locally or at the community ophthalmologist level. These patients could also perhaps benefit from earlier consideration of conjunctival revision. The finding that patch material thickness over the tube showed a significant group difference (*p* = 0.005) after six months, but total thickness (*p* = 0.067) did not, might suggest that mechanical stress, among other things, may be a factor in accelerating the reduction in thickness. It can be postulated that mechanical stress directly above the tube may be greatest during eye movement or blinking and that patch thinning would be particularly more significant at this site. Mechanisms such as retraction or compression of the patch material could not be detected in this cohort using solely Tutopatch^®^ pericardium. Patch retraction, dislocation, or mere compression would have been noticed during the revision surgery. One patient was also found to have had complete dissolution of the material without any residual patch tissue during revision surgery.

All patients received local application of MMC sponges as part of their surgery. The extent to which antimetabolites influence the patch material cannot be assessed based on the available data. This would require a comparison of patients without antimetabolite application or with the use of other substances. The use of MMC of course can have an influence on conjunctival health, thickness and healing responses, especially with prolonged and highly concentrated application, and thus increase the risk of erosion via mechanisms other than patch graft thinning. Current studies on AGVs indicate that the use of MMC has no influence on the long-term results after six months [17, 18]. Future studies on the PGI, which was used in our study, should also address this question, as the influence of MMC on the long-term outcome after implantation of a PGI has not yet been investigated.

Although the observation period of six months is relatively short, a significant number of tube erosions were already seen in this short period. In a study by Koh et al. on PGI outcomes, the erosion rate of 4.1% was significantly lower than in this study. However, the proportion of patients without previous surgery was higher in the study by Koh et al. and could possibly explain this difference [19]. The type of patch material used in patients with tube erosion was also not described, which could be an important influential factor. In a study just recently accepted for publication from our research group, and via the patient cohort described here, we demonstrate that the use of pericardium as the patch material of choice might not be ideal and other patch types could potentially be better in preventing erosion [20]. The Primary Tube Versus Trabeculectomy (PTVT) study by Gedde et al. also showed a significantly lower rate of tube erosion [1]. However, this study only included patients without incisional ocular surgery and is therefore only comparable with our study to a limited extent, as our study had no such exclusion criteria and included patients who had undergone complex prior surgery. In fact, previous ocular surgeries have been described as a known risk factor for tube erosion [21].

Different patch materials such as Tutopatch^®^ pericardium, fascia lata, sclera or human cornea presumably show different changes over time. Based on our data, pericardium does not appear to be the ideal material for covering the tube during GDI implantation in some patients. However, if pericardium is used to cover the tube, it should be used as a double-layered patch graft, as previous studies have shown a higher rate of erosion with single-layer use [22]. Similar results were obtained by van Hoefen Wijsard et al. who showed that human donor sclera was superior to bovine pericardium in terms of erosion rate, but without the use of AS-OCT [23]. Future studies should use AS-OCT imaging to investigate advantages and disadvantages between the different materials to define the most suitable materials. Furthermore, AS-OCT imaging should be used in the development of future materials. However, it should be noted that there might be limitations with regard to the penetrance and reflectivity properties of other materials. It can be assumed that very thick or non-transparent materials may be less suitable for an AS-OCT examination due to the limited penetration and image quality.

This study has several limitations including the rather small sample size. Larger numbers of patients in future studies could possibly reveal preoperative differences and risk factors that did not prove to be statistically significant in this study. In this way, patients with a higher probability of future erosion could be identified pre-operatively and other surgical techniques or patch materials could be considered. Moreover, various systemic diseases such as autoimmune diseases could have an influence on the rate of patch material breakdown through the release and activation of immune cells. An already known factor favouring the dissolution of patch materials and possible consecutive tube erosions is an existing ocular surface disease which we did not measure in our analysis [24]. Differences in tube size on the incidence of tube erosions could also not be investigated as we only looked at the PGI in this cohort. A future study including different tube types would be desirable.

## Conclusion

This study shows that AS-OCT is a useful method to follow-up patients who undergo patch graft implantation during PGI surgery. This method can potentially help identify patients at risk for implant exposure and could lead to modification of patient management to prevent implant exposure. Moreover, pericardium may not be the most suitable patch graft material. Future studies could utilize specific AS-OCT protocols to evaluate different types of patch characteristics including new patch materials.

## Declarations

All procedures performed in studies involving human participants were in accordance with the 1964 Helsinki Declaration and its later amendments or comparable ethical standards. The study was approved by the Ethics Committee of the University Hospital Bonn.
